# Neuromusculoskeletal Health in Pediatric Obesity: Incorporating Evidence into Clinical Examination

**DOI:** 10.1007/s13679-021-00463-9

**Published:** 2021-12-27

**Authors:** Grace C. O’Malley, Sarah P. Shultz, David Thivel, Margarita D. Tsiros

**Affiliations:** 1 School of Physiotherapy, Division of Population Health Sciences, RCSI University of Medicine and Health Sciences, Dublin, Ireland; 2 Child and Adolescent Weight Management Service, Children’s Health Ireland at Temple Street, Dublin, Ireland; 3 Kinesiology Department, Seattle University, Seattle, WA USA; 4 Laboratory of the Metabolic Adaptations To Exercise Under Physiological and Pathological Conditions, Auvergne Research Center for Human Nutrition (CRNH), Clermont Auvergne University, CEDEX 63000, UE3533 Clermont-Ferrand, AME2P France; 5 Alliance for Research in Exercise, Nutrition and Activity, UniSA Allied Health and Human Performance, University of South Australia, Adelaide, South Australia Australia; 6 Innovation, Implementation and Clinical Translation in Health (IIMPACT), UniSA Allied Health and Human Performance, University of South Australia, Adelaide, South Australia Australia

**Keywords:** Pediatric obesity, Musculoskeletal impairment, Personalized treatment, Rehabilitation, Activity limitation, Physiotherapy

## Abstract

***Purpose of Review*:**

The study aims to highlight the clinical importance of assessing and managing neuromusculoskeletal health in pediatric obesity and to support translation of evidence into practice.

***Recent Findings*:**

A growing evidence base suggests that children with obesity experience neuromusculoskeletal impairments and physical complications including increased pain, reduced muscle strength, impaired balance and motor skill, gait deviations, postural malalignment, greater fatigue, and potentially reduced flexibility and sub-optimal bone health. Such evidence supports the need to screen, assess, and optimize neuromusculoskeletal health as part of pediatric obesity management.

***Summary*:**

The likelihood of children with obesity experiencing neuromusculoskeletal impairments is high and can impact the way a child moves, and their interest or capacity to engage in physical activity and exercise. Barriers to movement should be minimized to promote optimal development of the neuromusculoskeletal system and to support engagement in sufficient physical activity for weight management. Healthcare professionals should screen for neuromusculoskeletal impairments as well as personalize interventions and modify standardized exercise interventions to optimize obesity treatment. Further research should explore whether neuromusculoskeletal impairments influence the success of obesity treatment or whether they improve following obesity treatment.

## Introduction

Mounting evidence suggests that childhood obesity may be associated with reduced physical functioning or ‘disability’ [1••] as defined by the WHO International classification of Functioning, Disability and Health (ICF). A recent umbrella review of 21 systematic reviews investigating 349 unique primary studies found ‘substantial impacts’ of childhood obesity on physical health that were most apparent as ICF impairments in body structures and function (e.g., neuromusculoskeletal impairment) [1••]. Positive cross-sectional association was evident between adiposity and musculoskeletal pain and musculoskeletal injury in children, whereas obesity inversely affected cardiorespiratory fitness, balance/coordination, general motor skills, functional mobility, and running speed/agility. Combined with an inverse association between adiposity and health-related quality of life, it becomes evident that carrying excess mass can have a significantly negative impact on a child’s ability to function, play, move, and engage in everyday life. Yet, these obesity-related impairments and complications are often not considered during clinical assessment, which could result in missteps when designing an effective personalized intervention plan or affect the child’s ability to adhere to the prescribed intervention.

Several high-quality systematic reviews have been published detailing the neuromusculoskeletal impairments observed in children and adolescents with obesity and the pathophysiological mechanisms involved. Building on these, this current report presents an overview of the impact of neuromusculoskeletal impairment and complications in children and adolescents with obesity and provides recommendations on how to translate the published evidence into clinical practice (e.g., short clinical encounters lasting 5–15 min).

## What Are the Neuromusculoskeletal Impairments and Complications of Childhood Obesity?

Common neuromusculoskeletal impairments associated with childhood obesity are summarized in Table 1. The likelihood of increased musculoskeletal pain is particularly pertinent, with evidence suggesting that children with obesity may be at a greater risk of overall musculoskeletal pain, with specific complaints in the spine or lower limb [1••, 2–4]. Possible mechanisms are unclear, although biomechanical deviations, altered pain perception, or low-grade inflammation linked with obesity have been proposed [1••]. Reduced lower limb muscle strength have been widely reported when strength is corrected for body mass or when children with obesity perform tasks that require movement or propulsion of their mass, thus highlighting likely functional strength deficits [5•, 6–8]. Evidence is also emerging of impaired balance [1••, 7, 9], in children with obesity, speculated to be linked with sensory deficits and/or relative muscle weakness. Similarly, children with obesity demonstrate gait deviations, spinal/lower-limb postural malalignment, motor skill impairment, greater fatigue, or perceived exertion [10–12]. Evidence is less clear on the impact of childhood obesity on reduced flexibility and impaired bone health [7, 9, 13–18].

**Table.1:** Common neuromusculoskeletal impairments and complications of childhood obesity

Complication	Recent key supporting evidence	Type of study
Increased pain (e.g., musculoskeletal pain, neck/back pain, lower limb pain)	Tsiros et al. [1••] Palmer et al. [3] Sanders et al. [2] Azabagic & Pranjic [4]	UR C SR C
Reduced lower limb muscle strength (relative to body mass or during mass-dependent tasks)	Tsiros et al. [1••] Rodrigues de Lima et al. [19] Garcia-Hermoso et al. [5•] Grao-Cruces et al. [6] Mahaffey et al. [7] Thivel et al. [8]	UR SR SR SR SR SR
Impaired balance (e.g., during challenging balance tasks involving a narrowed stance ± vision)*	Tsiros et al. [1••] Tsiros et al. [20•]* O’Malley et al. [21] Barnett et al. [22] Mahaffey et al. [7]	UR CS CS SR SR
Impaired coordination	Tsiros et al. [1••] Barnett et al. [22]	UR SR
Gait deviation (e.g., increased pelvic/hip/knee motion, prolonged stance phase, wider based gait)	Molina-Garcia et al. [15]	SR
Postural malalignment (increased lumbar lordosis, genu valgum, pes planus)	Molina-Garcia et al. [16•]	SR and MA
Flexibility (mixed/unclear evidence for reduced UL flexibility)	Mahaffey et al. [7]	SR
Reduced motor skill proficiency	Tsiros et al. [1••] Slotte et al. [18] Barnett et al. [22] Cattuzzo et al. [13] Mahaffey et al. [7]	UR SR SR SR SR

*C* cohort study, *CS* cross−sectional study, *SR* systematic review, *MA* meta−analysis, *UR* umbrella review

## How Might Neuromusculoskeletal Impairments and Complications Affect the Life of the Child?

In addition to neuromusculoskeletal impairment there is also increasing evidence that children with obesity may have a reduced ability to undertake specific functional tasks or activities (i.e., ICF ‘activity limitations’) [1••, 23]. Common limitations identified include difficulty with functional mobility (walk, crawl, run), reduced running speed/agility, and emerging evidence of difficulties with climbing stairs and getting up from a chair [1••, 24–26]. Adiposity-related limitations in gross motor skills have also been widely reported (five systematic reviews) [1••], reflecting challenges with locomotor (e.g., jumping and hopping) and ball skills. Importantly, children with obesity perceive a lower level of health and fitness that can be mismatched to measured health outcomes [27]. These actual and perceived functional deficits combine to limit activity in children with obesity.

Children with obesity typically do not meet current recommended levels of moderate-vigorous physical activity; these levels are lower in comparison to leaner peers when examined using objective measures [28, 29]. Qualitative systematic/scoping reviews have explored common barriers to physical activity, indicating that children with obesity may be turned off activity by a range of ‘physical factors’ [12, 30]. Injury has been identified as a barrier to activity [12, 30] and a new meta-analysis of prospective evidence shows that youth with a higher BMI may be at a greater risk of injuring themselves during sport (OR 1.18) [31]. In addition, children with obesity have a higher risk of fractures [32]. Physical discomfort in the form of joint pain or shortness of breath and fatigue can prevent activity participation [12, 30]. Moreover, increased fatigue and higher rates of perceived exertion during weight-bearing activity may influence the type of activities that engage children with obesity [12, 10, 11]. Perceiving themselves to be less athletically capable can prevent children with obesity from participating in activity [12, 30]. Experiencing failure during physical activity was highlighted by youth as a factor contributing to their weight status, and low motivation to participate in activity or sport [12].

Motor skill competence has been cross-sectionally and prospectively linked with physical activity behaviors in children of varying weight status, supporting a possible ‘proficiency barrier’ [9, 22, 33, 34]. For instance, De Meester et al. [33] found that children with high motor skill competence were ~ 2.5 times more likely to achieve physical activity guidelines than those with low motor skill competence, although weight status was unknown in their sample. Another study reported that higher perceived/actual motor skill competence was predictive of more physical activity in 3rd- to 4th-grade children of all weight status [34]. Similarly, difficulties with running, hopping, walking, or ‘feeling clumsy’ were associated with lower sport participation in 7–14-year-olds with obesity [35]. More recently, evidence is emerging that slower development of fitness increases the risk of overweight and obesity [36] in children.

Participation in meaningful life situations may also be negatively impacted by obesity (i.e., ICF ‘participation restrictions’). Children with obesity consistently report impaired physical health-related quality of life (p-HRQOL) [37], suggesting that involvement in typical childhood life situations is adversely impacted (e.g., joining physical education class or a sports team or showering independently) [1••]. Tsiros et al. [24] found that higher adiposity in 10–13-year-olds was associated with less time spent in community participation activities, predominantly comprising of leisure/recreation (*r* − 0.23). Increasing participation in physical activity forms the foundation of obesity management and prevention [38]; activity interventions do have positive effects on health biomarkers including fundamental motor skill [39], muscle strength and performance [8], cardiometabolic and cardiorespiratory health, and body composition [40••]. Yet, meta-analytical evidence has shown that physical activity-promoting interventions have ‘no effect’ on overall physical activity in this population [41]. Thus, there are clearly limitations with current approaches to activity prescription for children with obesity. Instead, personalized interventions may be needed to address movement-related barriers to break a cycle of sub-optimal engagement in fun physical activity.

**Fig. 1:**
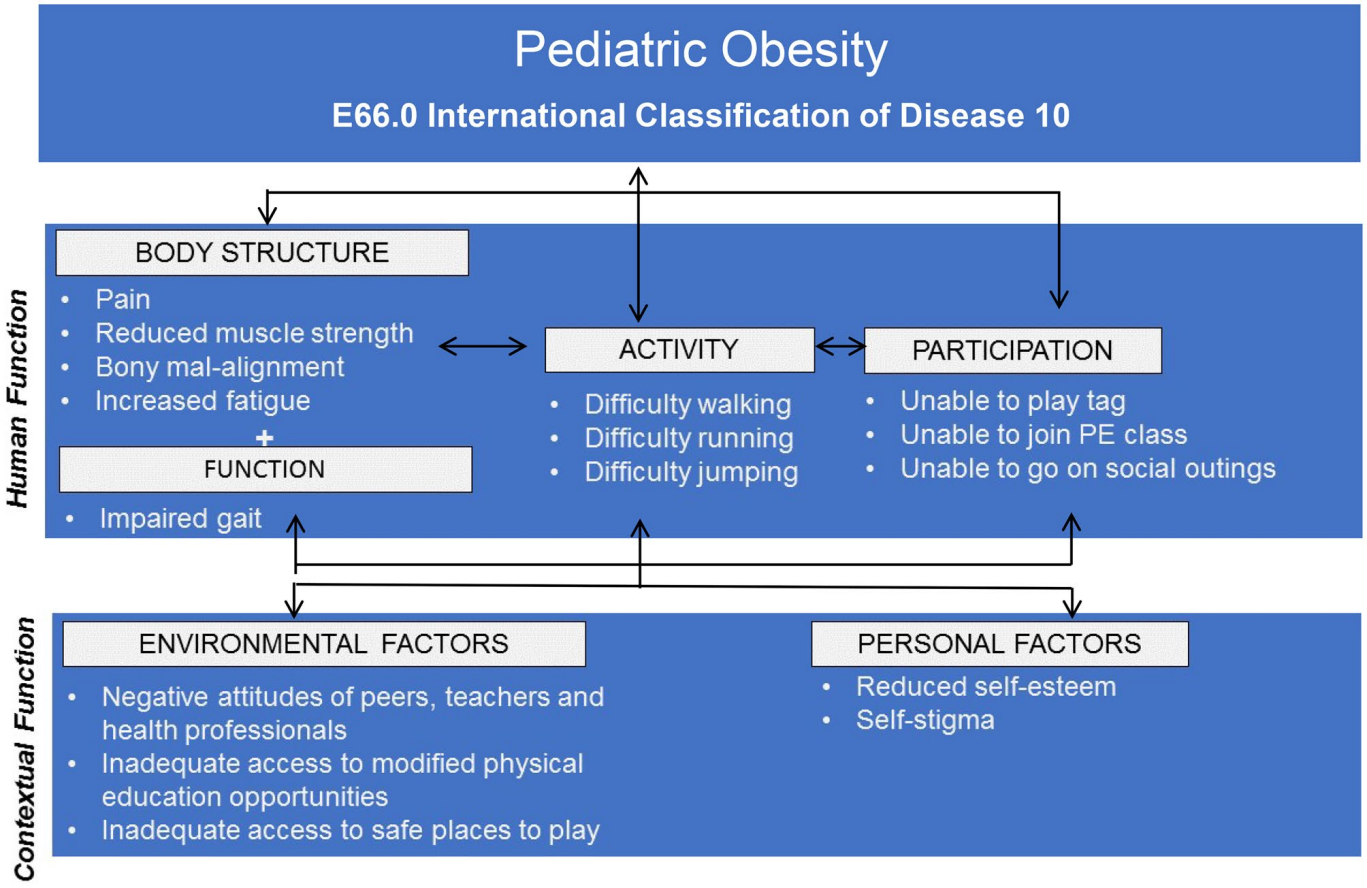
Interactions between the components of the ICF model with a pediatric obesity example

The ICF offers a theoretical framework for exploring such relationships, depicting complex, bidirectional interactions between body structure/function impairments, activity limitations, and participation restrictions [23]. For instance, it can be speculated that neuromusculoskeletal impairments such as pain, fatigue, reduced muscle strength, and fitness may mean that children with obesity do not have the necessary physical capabilities to participate in the typical physical activity and functional daily tasks expected of their peer group. A negative cycle of neuromusculoskeletal impairment, disability, lower physical competence, lower self-esteem, and reduced physical activity participation may ensue, further compounding weight status and associated functional deficits. Alternatively, physical fitness (comprising of strength, agility, and flexibility) was found to mediate the relationship between weight status and most HRQOL domains in children with overweight or obesity [42]. Both cardiorespiratory [42, 43] and muscular fitness (upper and lower limb) [42] have reported positive associations with physical wellbeing in children with obesity. Notably, these studies further suggest that negative associations between adiposity and physical wellbeing may be offset by muscular and/or cardiorespiratory fitness [42, 43]. Reduced lower extremity function (tests of single-leg balance and hop for distance) has also been associated with reduced p-HRQOL in children with obesity (explaining 48% variance) [44]. Evidence exploring such positive and negative links is beginning to emerge in the literature [9, 22, 33–35, 42–45]. Collectively, this emerging evidence suggests that certain obesity-related neuromusculoskeletal impairments and complications in ICF body function (e.g., single-leg balance, pain, bone strength, cardiorespiratory fitness, and physical fitness) may have negative links with physical wellbeing, which incorporates activities of daily living, play, and mobility as illustrated in Fig. 1. Moreover, restrictions in ICF activity (motor skills and isolated proficiency in running, hopping, walking) may be linked with lower physical activity and sport participation, increased injury, or impaired physical wellbeing. Thus, such factors may be potential assessment priorities when planning interventions for children and adolescents with obesity, although clearly more research is needed to confirm generalizability, causation, and probable bi-directional relationships. Thus, more quantitative research is needed to isolate the neuromusculoskeletal and movement-related factors that are likely to be most important in informing targeted interventions to enhance activity and participation in children with obesity.

## What Questions Can Illuminate Patient-Specific Challenges in Physical Function for the Child with Obesity?

Given the increased risk of neuromusculoskeletal impairment and subsequent activity limitation borne by children with obesity, it is essential that health professionals understand their role in screening, assessing, and addressing these complications (either through onward referral or direct treatment). *Assessment* should be conducted using open, respectful, and non-stigmatizing communication and a strength-based approach should value the existing knowledge, preferences, skills, and capacity of the child related to physical health, fitness, and participation. In the first instance screening questions (Table 2) can assist in triaging whether a child will require subsequent objective physical assessment, onward referral, or personalized intervention. Table 2 builds on previous suggestions for screening by the European Childhood Obesity Group [46] and can be a useful tool to those working in primary care to focus obesity assessment on outcomes other than body shape, size, or weight. Health professionals should listen carefully to how the parent and child respond to the questions throughout the clinical encounter. These questions should take no more than 5 mins. and thus, can easily be implemented into a standard wellness check. It should be noted that typical orthopedic variants occur throughout childhood development, and apophysites and osteochondroses are common causes of pain through adolescence [47, 48]. Therefore, health professionals should manage such conditions following current clinical recommendations [47, 48] and integrate into the overall obesity intervention. Health professionals should also be cognizant that movement difficulties may be impacted by additional obesity-related complications including impaired cardiorespiratory fitness, abdominal pain, hypertension, urinary incontinence, body consciousness or idiopathic intracranial hypertension and as such the neuromusculoskeletal assessment should be considered within the context of a holistic examination.

**Table.2:** Suggested questions addressing neuromusculoskeletal complications related to pediatric obesity

No	Question to parent and/or their child	Why ask?	What next?
1	Does your child/adolescent report any pain or discomfort in their feet, legs, back, arms, neck, or anywhere else?	Children with obesity experience more pain, particularly in the lower extremity which may influence engagement in active play/sport.	If present, thorough pain profiling and objective musculoskeletal assessment and treatment by a suitably qualified pediatric HCP is indicated.
2	Does your child/adolescent fatigue (get tired) easily when playing games/activities compared to others their age?	Children with obesity can experience greater muscle fatigue and perceived exertion especially in weight-bearing activity which can affect their ability or interest in playing actively with peers.	If present, objective musculoskeletal assessment, and sleep screening indicated. Treatment by a suitably qualified pediatric HCP is indicated.
3	Does your child/adolescent have difficulties staying balanced when moving/playing or do they fall more often compared to others their age?	Children with obesity can have more impaired balance and are more at risk of falling.	If present, objective assessment and treatment by a suitably qualified pediatric HCP is indicated.
4	Does your child/adolescent experience any physical difficulties with everyday childhood tasks (e.g., walking, running, jumping, hopping, ball skills, physical play, dressing, toileting, showering, picking something up from the floor)?	Children with obesity can have more impaired functional capacity and fundamental motor skills which can affect their ability or interest in playing actively with peers.	If present, objective assessment and treatment by a suitably qualified pediatric HCP is indicated.
5	Does your child/adolescent experience any physical difficulties with participating within the community (e.g. PE, cycling to school)?	Children with obesity can have more impaired coordination and fundamental motor skills which can affect their ability to engage in organized activities with their peers. Children with obesity may experience isolation due to physical impairments or increased risk of teasing by peers.	If present, objective assessment and treatment by a suitably qualified pediatric HCP is indicated.
6	Does your child/adolescent experience any other difficulties when moving, or playing (e.g., breathing difficulties, headaches, urinary incontinence, teasing/bullying)?	Children with obesity can have more impaired respiratory function, higher blood pressure, and greater risk of incontinence and bullying which can affect their functional capacity or interest in playing with peers.	If present, objective assessment and treatment by a suitably qualified pediatric HCP is indicated.
8	In the last week, how many days was your child engaged in moderate or vigorous physical activity for at least 60 min/day (‘huff and puff’ activity)?	Children with obesity can have lower levels of MVPA compared to lean peers. Understanding why a child is not participating in the recommended level is key to addressing these barriers through progressive physical activity intervention. Lower MVPA levels may be due to greater fatigue, more physical impairments, and challenges participating in suitable fun and rewarding activities.	If not meeting recommended age-appropriate guideline for MVPA, explore if there are barriers and commence goal setting and problem solving to address these.
9	Is there anything about your child’s physical movement or activity that you/they are concerned about or would like to improve?	Additional factors including preferences, goals, financial difficulty, safety, low confidence, lack of comfortable clothes/shoes that fit, inability to fit in shower at home/school, or negative body image may need to be considered and integrated into treatment planning.	If parent or child express concern, determine whether these can be addressed by you or whether onward referral may be required.

*HCP* healthcare professional, *MVPA* moderate to vigorous physical activity

**Fig. 2:**
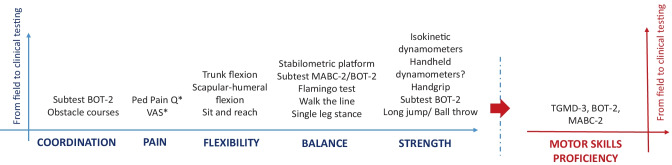
Non-exhaustive overview of the different tests that propose a reliable evaluation of each separate components and of the overall motor skill proficiency. *Thorough pain assessment required to elucidate type, frequency, location of pain plus aggravating/easing factors, and underlying cause of pain. *BOT-2* Bruininks-Oseretsky Test of Motor Proficiency 2nd Edition, *MABC-2* Movement Assessment Battery for Children 2nd Edition, *Ped Pain Q* PedsQL™ Pediatric Pain Questionnaire™, *TGMD-3* Test of Gross Motor Development 3rd Edition, *VAS* visual analogue scale (100 mm), *KTK* Körperkoordinations Test für Kinder.

For those treating children with severe pediatric obesity, a subsequent and more detailed clinical screening should be undertaken by health professionals who already have experience in treating physical impairment in children and adolescents with pediatric obesity and/or pediatric orthopedics. The screening questions described in Table 2 can be part of a more detailed physical examination using developmentally appropriate valid and reliable outcomes measures and tests. The type of outcome measure used will depend on the age of the child, the setting of the assessment and the availability of time and equipment. Figure 2 provides a non-exhaustive overview of the myriad tests used in assessing potential outcome measures of pediatric and adolescent neuromusculoskeletal health. Prior to undertaking neuromusculoskeletal assessment the need for, and procedures related to, the assessment should be clearly explained to the child and parent/s and appropriate consent obtained.

## How Do We Use the Knowledge Gained from Screening Questions and Objective Assessment to Develop an Appropriate Exercise Intervention?

Regardless of the physical capacity of the child or the existence of neuromusculoskeletal impairments, personalized physical activity and exercise interventions will be required as a cornerstone of obesity management. Similarly, the exercise intervention delivered as part of obesity management should be directed to address any observed neuromusculoskeletal impairments or complications (e.g., reduced strength) and improvement of such complications should be considered a successful outcome in obesity management. Table 3 describes some considerations and examples when designing exercise interventions for children and adolescents with obesity. The design of an appropriate exercise intervention will hinge on the findings of the physical assessment, the likelihood of impairments affecting engagement with treatment, and whether modifications to standard activity interventions may be required. The physical assessment will identify factors that should be addressed as part of holistic obesity management and will highlight the existence of red flags that should not be missed (e.g., night pain, multiple fractures, early morning joint stiffness, non-mechanical pain, unexplained muscle weakness/wasting, changes in sensation, regression of development, signs of slipped capital femoral epiphysis, skin alteration (bruising, psoriasis, café au lait), changes in bladder/bowel habit, concerns related to vision, speech or hearing). Health professionals may need to adapt or augment their ‘usual’ obesity exercise intervention to include non-weight bearing exercises, pacing techniques to address persistent pain or physical therapy modalities to treat specific impairments. For example, a child may present with medial knee pain that is exacerbated by loading during weight-bearing activity. In this example, it will be crucial to include the management of this pain as part of the obesity intervention to maximize engagement in physical activity and to support successful obesity management. The healthcare professional should clearly explain to patients and their parents that assessing and addressing neuromusculoskeletal impairments is important for supporting increased activity level and intensity as part of obesity treatment. Similarly, baseline assessment will support the health professional in commencing the fun activity intervention personalized to the child’s fitness level and preferences and taking into consideration any contraindications or precautions related to exercise. Thereafter a progressive program can be planned using collaborative goal setting and FITT-VP principles (frequency, intensity, time, type, volume, and progression) [49]. As the child progresses through treatment, monitoring of fatigue, pain, or other impairments identified during assessment should be undertaken and repeated measurement of baseline outcome measures can be integrated to evaluate how obesity treatment impacts physical fitness, function, and participation in tandem with measures of adiposity and cardiometabolic health. Given the increased risk of neuromusculoskeletal complications related to pediatric obesity, child-centered personalized treatment may also facilitate the prevention of musculoskeletal disease as the child grows (a leading cause of global disability) [50].

**Table 3:** Key considerations for exercise interventions in pediatric obesity management

No	Consideration	Action	Example
1	Identify pediatric contraindications to exercise.	Determine whether signs and symptoms contraindicate participation in an exercise intervention or whether the design or implementation of the exercise intervention should be modified based on identified red flags.	• Early morning stiffness and polyarticular pain identified on objective assessment. Further work-up should exclude juvenile idiopathic arthritis. Adaption to exercise intervention will be required to manage fatigue and joint pain. •Unilateral limp, reduced range of motion at hip and/or unilateral knee pain identified on objective assessment. Further work-up should exclude slipped capital femoral epiphysis (SCFE) and exercise intervention should be withheld until SCFE is excluded. • Neuromotor delay and increased tone noted during objective assessment. Further work-up should exclude neurological disease or developmental disorder. Adaption of exercise intervention will be needed to address any motor asymmetry, muscle spasm, or muscle weakness. • Fracture history should be noted, including mechanism of injury and if concerned further work-up to assess bone health and exclude osteopenia or other condition. Adaption to exercise intervention might include specific bone building exercises or avoidance of certain movements or contact sports that might increase risk of fracture.
2	Treatment planning of exercise intervention based on objective findings of clinical assessment.	Modify or adapt exercise intervention in line with baseline fitness and underlying health complications or impairments.	• Knee pain may require initial intervention using non-weight-bearing exercise or specific physical therapy intervention. • • Hypertension may necessitate paced increase in intensity and avoidance of exercises that precipitate valsalva maneuver. • Urinary incontinence may require specific physical therapy intervention addressing pelvic floor function, avoidance of high-impact activities, management with sanitary products, or onward referral.
3	Facilitate buy-in and understanding regarding the aim of the exercise intervention.	Explain how a tailored exercise intervention can address the strengths and impairments of the child identified during the clinical assessment phase.	Explain the benefit of increasing fitness for enhancing the activities and tasks the child is already good at and for improving confidence, insulin sensitivity, blood pressure, and body composition.
4	Planning of exercise intervention based on developmental, socioeconomic, and cultural context.	Design exercise intervention appropriate to child’s age, developmental stage, socioeconomic situation, and cultural considerations.	Child may not possess optimal fundamental motor skills needed for joining in with peers, may not be able to afford to join a gym/sports, or may be discouraged to exercise if female. Incorporation of training for fundamental motor skill will be required as part of exercise intervention.
5	Planning of exercise intervention around child’s preferences and social support available.	Optimize engagement with and adherence to exercise intervention by incorporating activities the child finds enjoyable and those for which support exists from family members, peers, teachers, or friends.	If child is nervous about team-based games/exercise, focus should be on active play and increasing levels of fun by letting the child suggest activities/games. Encourage parents/siblings/peers to support and play with child.
6	Consider body image, self-efficacy, confidence, and skin chafing.	Optimize adherence to the exercise intervention by addressing negative body image, low self-efficacy, or low moto-r-confidence if present. Address skin chafing if present.	Incorporate activities to optimize posture, balance, and confidence in movement. Encourage child/adolescents to wear comfortable clothing and underwear (e.g. cotton sports bra) that support movement. Advise on use of talcum powder and petroleum jelly if appropriate.
7	Address fear of falls if present.	Assess whether and how child can get up independently if they fall or are playing on the floor.	Teach backward chaining* to encourage independence and confidence around getting up from floor.
8	Use goal setting to plan child-centered exercise intervention.	Plan specific, measurable, achievable, realistic, and time-based goals with the child and parent/s to build physical function and physical fitness. Consider family’s usual routine and aims of the child.	Child may first aim to walk to school without discomfort, meeting peers/siblings for outdoor play or improve ball skills followed by participating more in physical education class, learning to ride a bicycle, and joining organized sports or activities. Family aims to play/conduct the prescribed games/activities for 30 mins. with the child twice per week (Wed and Sat) in addition to bringing to supervised exercise session twice per week (Mon and Fri).
9	Reduce sedentary time.	Assess the time child spends using screens for entertainment, sitting throughout the day, and number of movement breaks.	Educate family and child about importance of breaking up and reducing sedentary time where possible.
10	Use FITT-VP.	Use baseline assessment and fitness level to determine the frequency, intensity, time, type, volume, and progression of the exercise intervention.	If the child has severe obesity, low aerobic fitness, low musculoskeletal fitness, low activity levels, and high sedentary time, commence exercise intervention with shorter bouts of low intensity preferred activity 2–3 times per week, aiming to build up time, intensity, and variety of exercise.
11	Decide on metrics/outcomes to evaluate exercise intervention.	Consider the length of intervention and what health outcomes can realistically change within that time. Ensure family are aware of these in addition to planned impact on body composition.	Aim of intervention might be to walk to school without pain or fatigue, to reduce blood pressure, to increase strength of quadriceps, to improve standing balance, or to reduce musculoskeletal pain with more intense activity/exercise.

*Backward-chaining breaks a particular movement task down into steps. In the case of falls, the patient starts learning the task of getting up from the floor back up into standing or sitting on a chair. The patient starts in the most stable position and only progresses to more unstable positions (on knees, sitting, or lying on floor) as they are able

## Conclusion

Children and adolescents with obesity have higher risk of neuromusculoskeletal impairment which may influence their engagement with obesity interventions and in turn influence the design of effective obesity interventions. Health professionals should screen for such impairment as part of standard clinical assessment. In this paper we provide tools to support how health professionals can assess, address, adapt, progress, monitor, and evaluate the impact of treatment on neuromusculoskeletal health in children with obesity.

## Data Availability

Not applicable.
